# Hypaphorine Attenuates Lipopolysaccharide-Induced Endothelial Inflammation via Regulation of TLR4 and PPAR-γ Dependent on PI3K/Akt/mTOR Signal Pathway

**DOI:** 10.3390/ijms18040844

**Published:** 2017-04-17

**Authors:** Haijian Sun, Xuexue Zhu, Weiwei Cai, Liying Qiu

**Affiliations:** Department of Basic Medicine, Wuxi School of Medicine, Jiangnan University, Wuxi 214122, China; haijsunjiangnan@jiangnan.edu.cn (H.S.); baojiangexz@sina.com (X.Z.); hlxuzhou11@sina.com (W.C.)

**Keywords:** inflammation, endothelial cells, LPS, PPAR-γ, TLR4

## Abstract

Endothelial lesion response to injurious stimuli is a necessary step for initiating inflammatory cascades in blood vessels. Hypaphorine (Hy) from different marine sources is shown to exhibit anti-inflammatory properties. However, the potential roles and possible molecular mechanisms of Hy in endothelial inflammation have yet to be fully clarified. We showed that Hy significantly inhibited the positive effects of lipopolysaccharide (LPS) on pro-inflammatory cytokines expressions, including tumor necrosis factor-α (TNF-α), interleukin-1β (IL-1β), monocyte chemoattractant protein 1 (MCP-1) and vascular cellular adhesion molecule-1 (VCAM-1), as well as induction of the phosphorylation of Akt and mTOR in HMEC-1 cells. The downregulated peroxisome proliferator-activated receptor γ (PPAR-γ) and upregulated toll-like receptor 4 (TLR4) expressions in LPS-challenged endothelial cells were prevented by Hy. Inhibition of both PI3K and mTOR reversed LPS-stimulated increases in TLR4 expressions and decreases in PPAR-γ levels. Genetic silencing of TLR4 or PPAR-γ agonist pioglitazone obviously abrogated the levels of pro-inflammatory cytokines in LPS-treated HMEC-1 cells. These results suggest that Hy may exert anti-inflammatory actions through the regulation of TLR4 and PPAR-γ dependent on PI3K/Akt/mTOR signal pathways. Hy may be considered as a therapeutic agent that can potentially relieve or ameliorate endothelial inflammation-associated diseases.

## 1. Introduction

The vascular endothelium is a dynamic barrier between blood and blood vessel walls [1]. The endothelium is critically involved in vascular homeostasis via producing various vasoactive mediators including nitric oxide, adhesion molecules, growth factors, prostanoids, cytokines, and endothelins [2]. Chronic inflammation in endothelial cells produces a variety of inflammatory mediators to exacerbate endothelial dysfunction [3]. Endothelial dysfunction from inflammation plays a dominant role in atherosclerosis, hypertension and diabetes-induced vasculopathy and vascular remodeling [4,5]. Endothelial inflammation is also taken as a high-risk factor for cardiovascular adverse events [6,7].

Toll-like receptors (TLRs) are considered to be key participants in tissue damage and inflammation [8]. Activation of TLRs triggers an adaptive immune response, which includes cytokine production [8]. Among TLRs, lipopolysaccharides (LPSs) from Gram-negative bacteria interact with TLR4 to induce an inflammatory response in endothelial cells [9]. The expression of TLR4 is increased in atherosclerotic plaques of animal models, and atherosclerosis-associated inflammation is alleviated in TLR4 knockout mice [10]. Peroxisome proliferator-activated receptors (PPARs) belong to the nuclear receptor super family members that exert fundamental roles in cardiovascular protection [11]. PPAR-γ appears to be highly expressed in atherosclerotic lesions, and PPAR-γ activation efficiently ameliorates inflammatory response in cardiovascular cells, particularly in endothelial cells [12]. PPAR-γ ligands improve endothelial cell function via attenuating inflammation in diabetes and atherosclerosis [13]. PPAR-γ activation accelerates the apoptosis of esophageal cancer cells by the inhibition of the TLR4 pathway [14]. Chronic activation of TLR4 suppresses PPAR-γ transactivation to block brown adipogenesis of multipotent mesodermal stem cells and brown pre-adipocytes [15]. Crosstalk between TLR4 and PPAR-γ pathways contributes to the arachidonic acid-induced inflammatory response in pancreatic acini [16]. This current research establish the potential relationship between TLR4 and PPAR-γ in various biological functions.

Hypaphorine (Hy) is an indole alkaloid from *Erythrina velutina*, which promotes sleep effects in normal mice [17]. Hy from different marine sources exhibits anti-inflammatory properties [18]. However, whether and how Hy is involved in endothelial inflammation remains largely unclear. In this study, we investigated the effects of Hy on LPS-induced inflammation response, and we further explored whether TLR4 and PPAR-γ are involved in the potential roles of Hy in endothelial inflammation response to LPS. 

## 2. Results 

### 2.1. Effects of Hy on the Expressions of TNF-α, IL-1β, VCAM-1 and MCP-1 in HMEC-1 Cells Response to LPS

**Figure 1:**
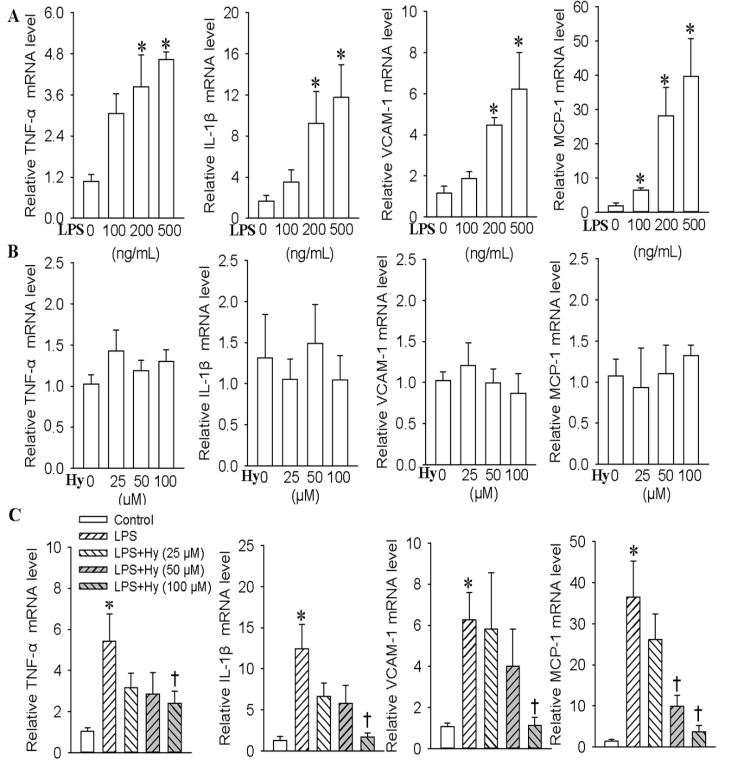
Effects of different doses of Hy on the mRNA expressions of TNF-α, IL-1β, VCAM-1 and MCP-1 response to LPS-treated HMEC-1 cells in vitro. (**A**) Effects of various concentrations of LPS (0, 100, 200 and 500 ng/mL for 48 h) on the TNF-α, IL-1β, VCAM-1 and MCP-1 levels in HMEC-1 cells; (**B**) Effects of different doses of Hy (0, 25, 50 and 100 μM for 48 h) on the mRNA expressions of TNF-α, IL-1β, VCAM-1 and MCP-1 HMEC-1 cells; (**C**) Effects of different doses of Hy (0, 25, 50 and 100 μM) on the TNF-α, IL-1β, VCAM-1 and MCP-1 expressions in LPS (500 ng/mL)-stimulated HMEC-1 cells. The HMEC-1 cells were pretreated with different doses of Hy for 6 h before LPS incubation for another 48 h. Values are mean ± S.D. * *p* < 0.05 vs. 0 ng/mL, 0 μM or control, † *p* < 0.05 vs. LPS. *n* = 4 for each group. LPS, lipopolysaccharide; TNF-α, tumor necrosis factor-α; IL-1β, interleukin-1β; VCAM-1, vascular cellular adhesion molecule-1; MCP-1, monocyte chemoattractant protein 1.

Vascular adhesion molecules and inflammatory cytokines are overexpressed in activated endothelial cells [19,20]. First, we examined whether Hy (Figure S1) protected HMEC-1 cells against LPS-mediated inflammation. Incubation of HMEC-1 cells with LPS resulted in a dose-dependent stimulation of the pro-inflammatory cytokines expressions of TNF-α (3.8-fold for 200 ng/mL, *p* < 0.05, 4.6-fold for 500 ng/mL, *p* < 0.05), IL-1β (9.2-fold for 200 ng/mL, *p* < 0.05, 11.7-fold for 500 ng/mL, *p* < 0.05), MCP-1 (6.4-fold for 100 ng/mL, *p* < 0.05, 28.1-fold for 200 ng/mL, *p* < 0.05, 39.7-fold for 500 ng/mL, *p* < 0.05) and VCAM-1 (4.4-fold for 200 ng/mL, *p* < 0.05, 6.2-fold for 500 ng/mL, *p* < 0.05) (Figure 1A). However, different concentrations of Hy had no obvious effect on TNF-α, IL-1β, MCP-1 and VCAM-1 levels (Figure 1B). It is interesting that Hy at higher dose (100 μM) abolished the upregulated expressions of TNF-α (decreased by 55.6% for 100 μM at mRNA level, *p* < 0.05; decreased by 33.8% for 100 μM at protein level, *p* < 0.05), IL-1β (decreased by 86.3% for 100 μM at mRNA level, *p* < 0.05; decreased by 31.7% for 100 μM at protein level, *p* < 0.05), MCP-1 (decreased by 72.5% for 50 μM at mRNA level, *p* < 0.05, decreased by 91.6% for 100 μM at mRNA level, *p* < 0.05; decreased by 36.8% for 100 μM at protein level, *p* < 0.05) and VCAM-1 (decreased by 82.2% for 100 μM at mRNA level, *p* < 0.05; decreased by 26.4% for 100 μM at protein level, *p* < 0.05) induced by LPS in HMEC-1 cells (Figure 1C and Figure S2).

### 2.2. Inhibition of TLR4 Was Involved in the Anti-Inflammatory Action of Hy on HMEC-1 Cells Response to LPS 

**Figure 2:**
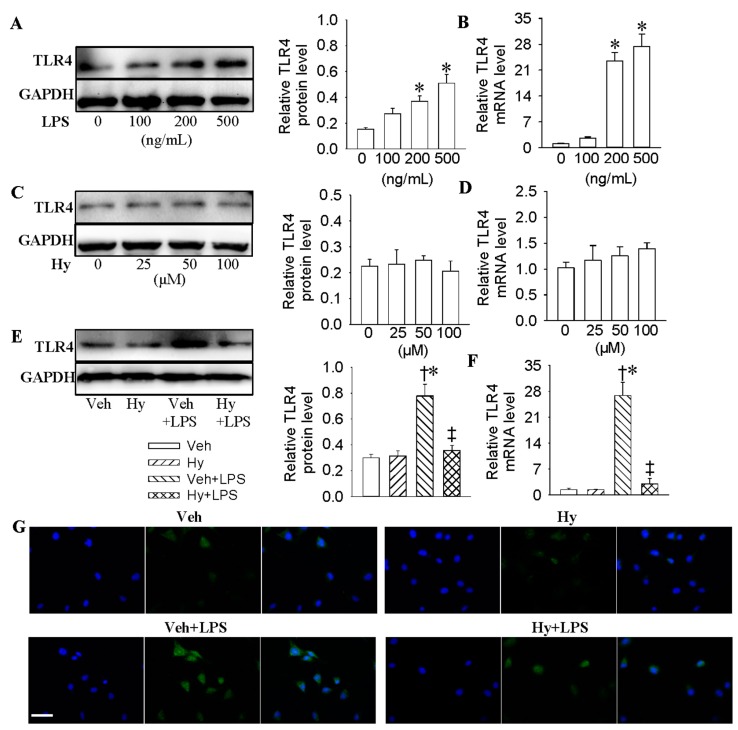
Inhibition of TLR4 was involved in the anti-inflammatory action of Hy on HMEC-1 cells response to LPS. (**A**) Representative photos showing the effects of various concentrations of LPS (0, 100, 200 and 500 ng/mL for 48 h) on TLR4 protein levels in HMEC-1 cells; (**B**) Effects of various concentrations of LPS (0, 100, 200 and 500 ng/mL for 48 h) on TLR4 mRNA levels in HMEC-1 cells; (**C**) Effects of different doses of Hy (0, 25, 50 and 100 μM for 48 h) on TLR4 protein levels in HMEC-1 cells; (**D**) Effects of different doses of Hy (0, 25, 50 and 100 μM for 48 h) on TLR4 mRNA levels in HMEC-1 cells; (**E**) Representative photographs showing the effects of Hy (100 μM) on TLR4 protein expressions in LPS (500 ng/mL)-challenged HMEC-1 cells. The HMEC-1 cells were pretreated with Hy for 6 h before LPS incubation for another 48 h; (**F**) Effects of Hy (100 μM ) on TLR4 mRNA expressions in LPS (500 ng/mL)-challenged HMEC-1 cells; (**G**) Immunofluorescence staining showing the effects of Hy (100 μM ) on TLR4 protein expressions in HMEC-1 cells response to LPS (500 ng/mL), scale bar = 50 µm. Values are mean ± S.D. * *p* < 0.05 vs. 0 ng/mL or Veh, † *p* < 0.05 vs. Hy, ‡ *p* < 0.05 vs. Veh + LPS. *n* = 4 for each group. LPS, lipopolysaccharide; TLR4, toll-like receptor 4.

A variety of studies have shown that TLR4 activation is majorly responsible for the initiation of proinflammatory responses in endothelial cells [21,22,23]. We investigated whether the inhibitory effects of Hy on inflammatory mediators production was related to the modulation of TLR4. LPS strongly stimulated TLR4 expression at both protein (1.6-fold for 200 ng/mL, *p* < 0.05, 2.4-fold for 500 ng/mL, *p* < 0.05) and mRNA (23.4-fold for 200 ng/mL, *p* < 0.05, 27.4-fold for 500 ng/mL, *p* < 0.05) levels in a dose-dependent manner (Figure 2A,B), whereas Hy exhibited no effect on the protein and mRNA expressions of TLR4 (Figure 2C,D). Western blot results showed that Hy effectively suppressed the protein (decreased by 53.8% for 100 μM, *p* < 0.05) and mRNA (decreased by 84.2% for 100 μM, *p* < 0.05) expressions of TLR4 induced by LPS (Figure 2E,F). The inhibitory effects of Hy on TLR4 levels in LPS-stimulated HMEC-1 cells were also demonstrated by immunofluorescence staining (Figure 2G and Figure S3). Furthermore, the inhibition of TLR4 with siRNA markedly prevented the expressions of TNF-α (decreased by 60.3% at mRNA level, *p* < 0.05; decreased by 38.9% at protein level, *p* < 0.05), IL-1β (decreased by 82.9% at mRNA level, *p* < 0.05; decreased by 34.1% at protein level, *p* < 0.05), MCP-1 (decreased by 67.1% at mRNA level, *p* < 0.05; decreased by 29.1% at protein level, *p* < 0.05) and VCAM-1 (decreased by 54.1%, *p* < 0.05; decreased by 24.5% at protein level, *p* < 0.05) in LPS-incubated HMEC-1 cells (Figure S4). 

### 2.3. Activation of PPAR-γ Mediated the Protective Role of Hy against LPS-Evoked Inflammation in HMEC-1 Cells 

**Figure 3:**
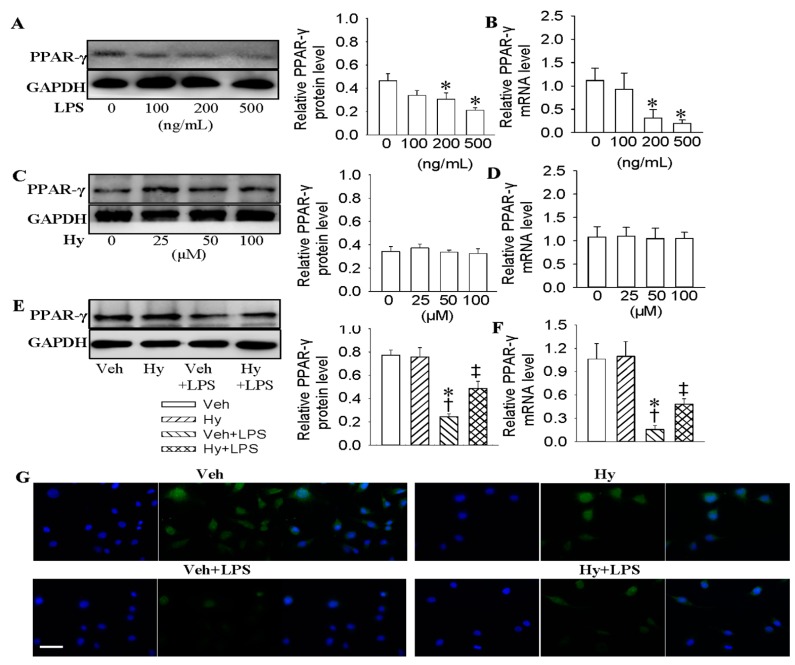
Activation of PPAR-γ mediated the protective role of Hy against LPS-evoked inflammation in HMEC-1 cells. (**A**) Representative photos showing the effects of various concentrations of LPS (0, 100, 200 and 500 ng/mL for 48 h) on PPAR-γ protein levels in HMEC-1 cells; (**B**) Effects of various concentrations of LPS (0, 100, 200 and 500 ng/mL for 48 h) on PPAR-γ mRNA levels in HMEC-1 cells; (**C**) Effects of different doses of Hy (0, 25, 50 and 100 μM for 48 h) on PPAR-γ protein levels in HMEC-1 cells; (**D**) Effects of different doses of Hy (0, 25, 50 and 100 μM for 48 h) on PPAR-γ mRNA levels in HMEC-1 cells; (**E**) Representative photographs showing the effects of Hy (100 μM) on PPAR-γ protein expressions in LPS (500 ng/mL)-challenged HMEC-1 cells. The HMEC-1 cells were pretreated with Hy for 6 h before LPS incubation for another 48 h; (**F**) Effects of Hy (100 μM) on PPAR-γ mRNA expressions in LPS (500 ng/mL)-challenged HMEC-1 cells; (**G**) Immunofluorescence staining showing the effects of Hy (100 μM) on PPAR-γ expressions in HMEC-1 cells response to LPS (500 ng/mL), scale bar = 50 μm. Values are mean ± S.D. * *p* < 0.05 vs. 0 ng/mL or Veh, † *p* < 0.05 vs. Hy, ‡ *p* < 0.05 vs. Veh + LPS. *n* = 4 for each group. LPS, lipopolysaccharide; PPAR-γ, peroxisome proliferator-activated receptor γ.

The decreased PPAR-γ is a critical determinant for ox-LDL-induced endothelial dysfunction [24]. Endothelial PPAR-γ is recognized as a potential candidate for vascular protection from atherosclerosis [25]. To confirm whether PPAR-γ was involved in the protective effects of Hy against LPS-induced inflammation, the protein and mRNA levels of PPAR-γ were determined. LPS dose-dependently decreased the protein (decreased by 32.6% for 200 ng/mL, *p* < 0.05, decreased by 54.3% for 500 ng/mL, *p* < 0.05) and mRNA (decreased by 69.2% for 200 ng/mL, *p* < 0.05, decreased by 82.3% for 500 ng/mL, *p* < 0.05) levels of PPAR-γ (Figure 3A,B), but Hy had no obvious effect on either protein or mRNA levels of PPAR-γ (Figure 3C,D). Nevertheless, Hy rescued both the protein (2.1-fold for 100 μM, *p* < 0.05) and mRNA (3.3-fold for 100 μM, *p* < 0.05) expression of PPAR-γ in LPS-treated HMEC-1 cells (Figure 3E,F). Immunofluorescence results further demonstrated that the reduced PPAR-γ expressions in LPS-challenged HMEC-1 cells were reversed by Hy (Figure 3G and Figure S3). In addition, PPAR-γ agonist pioglitazone significantly attenuated the overexpression of TNF-α (decreased by 60.7% at mRNA level, *p* < 0.05; decreased by 37.8% at protein level, *p* < 0.05), IL-1β (decreased by 56.1% at mRNA level, *p* < 0.05; decreased by 37.1% at protein level, *p* < 0.05), MCP-1 (decreased by 73.8% at mRNA level, *p* < 0.05; decreased by 22.5% at protein level, *p* < 0.05) and VCAM-1 (decreased by 54.1% at mRNA level, *p* < 0.05; decreased by 23.4% at protein level, *p* < 0.05) in LPS-pretreated cells (Figure S5). 

### 2.4. Interaction of PPAR-γ with TLR4 in HMEC-1 Cells in Response to LPS

**Figure 4:**
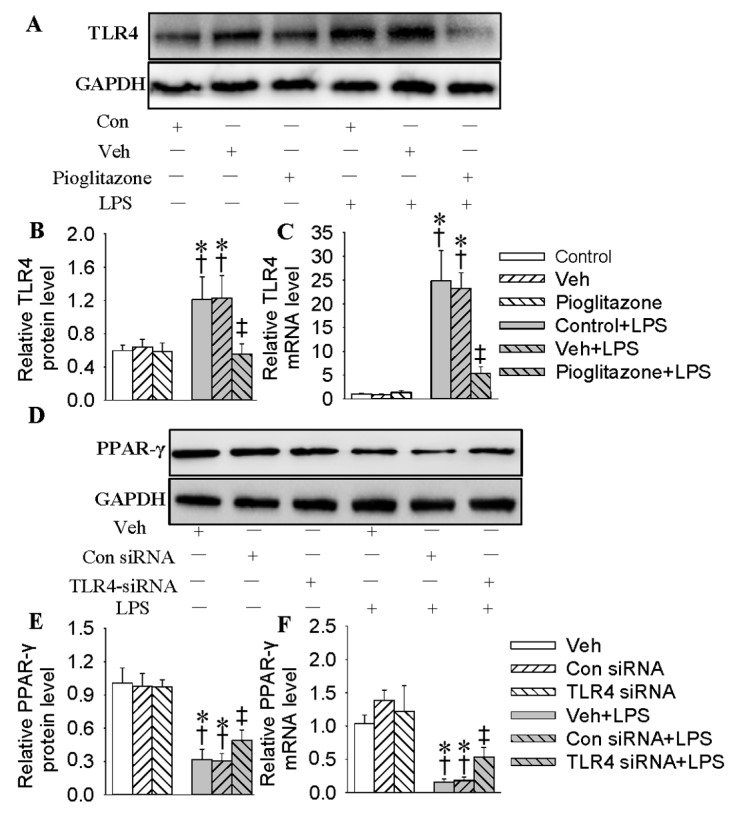
Interaction of PPAR-γ with TLR4 in HMEC-1 cells in response to LPS. (**A**) Western blotting of TLR4 in HMEC-1 cells. The HMEC-1 cells were pre-incubated with pioglitazone (20 μM) for 6 h followed by LPS (500 ng/mL) stimulation for 48 h; (**B**) Relative density of TLR4 protein bands determined by densitometry of the blots, (**C**) TLR4 mRNA levels; (**D**) Western blotting of PPAR-γ in HMEC-1 cells. The HMEC-1 cells were transfected with 100 nM control siRNA or TLR4 siRNA for 24 h followed by LPS (500 ng/mL) stimulation for 48 h; (**E**) Relative density of PPAR-γ protein bands determined by densitometry of the blots; (**F**) PPAR-γ mRNA levels. Values are mean ± S.D. * *p* < 0.05 vs. control or Veh, † *p* < 0.05 vs. Con(control) siRNA, ‡ *p* < 0.05 vs. control + LPS or Veh + LPS. *n* = 4 for each group. LPS, lipopolysaccharide; TLR4, toll-like receptor 4, PPAR-γ, peroxisome proliferator-activated receptor γ.

We hypothesized that TLR4 and PPAR-γ interaction may participate in LPS-induced inflammation. PPAR-γ activation had no effect on basal TLR4 in quiescent HMEC-1 cells (Figure 4A,B). Pretreatment with pioglitazone abolished TLR4 expressions at both protein (decreased by 54.4%, *p* < 0.05, Figure 4A,B) and mRNA (decreased by 78.2%, *p* < 0.05, Figure 4C) levels from LPS-stimulated HMEC-1 cells. Knockdown of TLR4 with siRNA effectively downregulated the protein and mRNA levels of TLR4 in HMEC-1 cells (Figure S6). Reduction of TLR4 remarkably interrupted the downregulated PPAR-γ protein (1.6-fold, *p* < 0.05, Figure 4D,E) and mRNA (3.3-fold, *p* < 0.05, Figure 4F) levels in HMEC-1 cells response to LPS. It is interesting that the activation of PPAR-γ with pioglitazone not only abated the basal TLR4 expression (decreased by 56.6%, *p* < 0.05) in quiescent cells, but also tremendously compromised LPS-induced TLR4 expression (decreased by 72.4%, *p* < 0.05) in EA.hy926 cells (Figure S7). Moreover, GW9662, a PPAR-γ antagonist, blocked pioglitazone-mediated inhibition of TLR4 in LPS-challenged HMEC-1 cells (Figure S8). 

### 2.5. PI3K/Akt/mTOR Signaling Pathway Was Responsible for the Inhibitory Effect of Hy on LPS-Induced Inflammation Response in HMEC-1 Cells 

**Figure 5:**
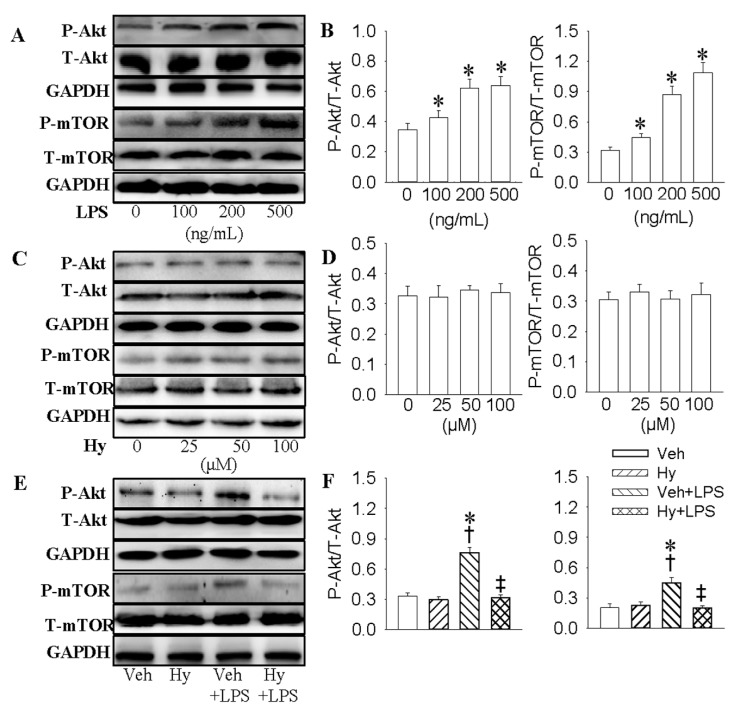
PI3K/Akt/mTOR signaling pathway is involved in the inhibition effect of Hy on LPS-induced inflammation response in HMEC-1 cells. (**A**) Effects of LPS on the protein expressions of total Akt (T-Akt), phosphorylated-Akt (p-Akt), mTOR, and phosphorylated-mTOR (p-mTOR) at indicated doses (0, 100, 200 and 500 ng/mL) in HMEC-1 cells. The confluent HMEC-1 cells were stimulated with different doses of LPS (0, 100, 200 and 500 ng/mL) for 15 min; (**B**) Quantitative analysis of phosphorylated Akt and mTOR; (**C**) Effects of Hy on the expressions of Akt, p-Akt, mTOR, and p-mTOR at indicated doses (0, 25, 50 and 100 μM) in HMEC-1 cells. The HMEC-1 cells were starved for 12 h and then stimulated with different doses (0, 25, 50 and 100 μM) of Hy for 15 min; (**D**) Quantitative analysis of phosphorylated Akt and mTOR; (**E**) Effects of Hy on phosphorylated Akt and mTOR in HMEC-1 cells response to LPS. The HMEC-1 cells were pretreated with Hy for 6 h before LPS incubation for 15 min; (**F**) Quantitative analysis of phosphorylated Akt and mTOR protein bands determined by densitometry of the blots. Values are mean ± S.D. * *p* < 0.05 vs. 0 ng/mL or Veh, † *p* < 0.05 vs. Hy, ‡ *p* < 0.05 vs. Veh + LPS. *n* = 4 for each group. LPS, lipopolysaccharide; TNF-α, tumor necrosis factor-α; IL-1β, interleukin-1β; VCAM-1, vascular cellular adhesion molecule-1; MCP-1, monocyte chemoattractant protein 1.

Blockade of PI3K/Akt/mTOR signaling pathway represses senescence-associated inflammation in endothelial cells [26]. Mango polyphenolics diminishes inflammation in intestinal colitis by modulating the PI3K/Akt/mTOR pathway [27]. To investigate the roles of PI3K/Akt/mTOR signaling in an LPS-mediated inflammation cascade, HMEC-1 cells were treated with PI3K inhibitor LY294002 or mTOR inhibitor rapamycin, respectively. Incubation of HMEC-1 cells with LPS significantly enhanced the phosphorylated Akt (1.2-fold for 100 ng/mL, *p* < 0.05, 1.8-fold for 200 ng/mL, *p* < 0.05, 1.9-fold for 500 ng/mL, *p* < 0.05) and mTOR (1.4-fold for 100 ng/mL, *p* < 0.05, 2.7-fold for 200 ng/mL, *p* < 0.05, 3.4-fold for 500 ng/mL, *p* < 0.05, Figure 5A,B). Hy had no significant effect on the phosphorylated Akt and mTOR (Figure 5C,D), but dramatically counteracted the phosphorylated Akt (decreased by 57.9%, *p* < 0.05) and mTOR (decreased by 55.6%, *p* < 0.05) in HMEC-1 cells response to LPS (Figure 5E,F). Moreover, both LY294002 and rapamycin eliminated the overproduction of TNF-α (decreased by 73.4% and 55.1% at mRNA level, *p* < 0.05; decreased by 37.1% and 29.2% at protein level, *p* < 0.05), IL-1β (decreased by 75.9% and 82.1% at mRNA level, *p* < 0.05; decreased by 27.3% and 33.6% at protein level, *p* < 0.05), MCP-1 (decreased by 65.6% and 59.3% at mRNA level, *p* < 0.05; decreased by 32.1% and 33.7% at protein level, *p* < 0.05) and VCAM-1 (decreased by 66.1% and 50.7% at mRNA level, *p* < 0.05; decreased by 38.2% and 27.3% at protein level, *p* < 0.05) induced by LPS (Figure S9).

### 2.6. Negative Correlation of TLR4 and PPAR-γ in LPS-Stimulated HMEC-1 Cells Was Dependent on the PI3K/Akt/mTOR Signaling Pathway 

**Figure 6:**
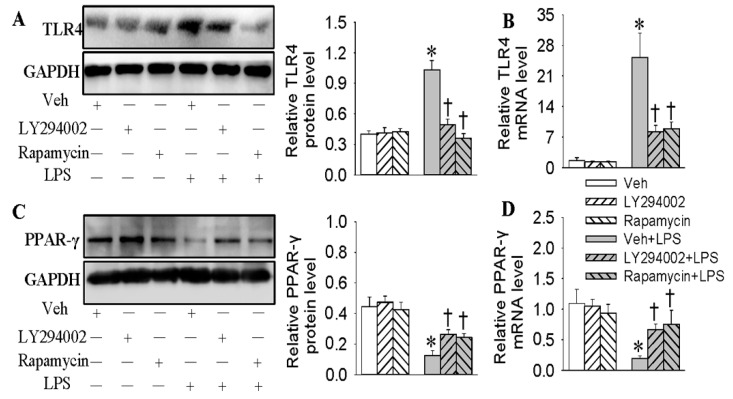
Negative correlation of PPAR-γ with TLR4 in LPS-stimulated HMEC-1 cells was dependent on PI3K/Akt/mTOR signaling pathway. The HMEC-1 cells were pretreated with LY294002 (10 μM), or mTOR inhibitor rapamycin (200 nM) for 6 h before LPS incubation for 48 h. The protein expressions of TLR4 (**A**) and PPAR-γ (**C**) were detected by Western blotting analysis. The mRNA expressions of TLR4 (**B**) and PPAR-γ (**D**) were detected by real time quantitative PCR. Values are mean ± S.D. * *p* < 0.05 vs. Veh, † *p* < 0.05 vs. Veh + LPS. *n* = 4 for each group. LPS, lipopolysaccharide; TLR4, toll-like receptor 4, PPAR-γ, peroxisome proliferator-activated receptor γ.

Finally, we wanted to examine whether the PI3K/Akt/mTOR signaling pathway modulated the relationship between PPAR-γ and TLR4 in LPS-treated HMEC-1 cells. The results showed that the inhibition of the PI3K/Akt/mTOR signaling pathway prevented both protein and mRNA levels of TLR4 (decreased by 51.4% and 65.1%, *p* < 0.05, Figure 6A,B) and PPAR-γ (2.2-fold and 2.1-fold, *p* < 0.05, Figure 6C,D). We confirmed that the negative correlation of TLR4 with PPAR-γ in LPS-incubated HMEC-1 cells was closely related with the PI3K/Akt/mTOR signaling pathway. 

## 3. Discussion

Accumulated evidence indicates that endothelial inflammation plays an important role in endothelial dysfunction and accelerates the development and progression of atherosclerosis [28]. In the present study, our results showed that Hy exerted a protective role against LPS-induced inflammation response in HMEC-1 cells, and that Hy may potentially serve as a protective candidate against LPS-induced inflammation in endothelial cells. 

Activation of endothelial cells stimulates the expression of adhesion molecules and proinflammatory cytokines, which is essential for plaque rupture and occlusive event in atherosclerosis [29]. In the present study, our results displayed that LPS dramatically upregulated pro-inflammatory cytokines expressions of TNF-α, IL-1β, MCP-1 and VCAM-1, while pretreatment with Hy abolished the increased expressions of TNF-α, IL-1β, MCP-1 and VCAM-1 induced by LPS in HMEC-1 cells. These results suggested that Hy-mediated anti-inflammatory effects offered a therapeutic strategy for the management of endothelial inflammatory diseases. 

TLRs are abundantly expressed in a plethora of cell types, such as endothelial cells [30,31]. A growing body of evidence suggests that TLR4 modulates endothelial inflammation and atherosclerotic disease [32]. LPS stimulates IL-6, MCP-1 and VCAM-1 mRNA expression in endothelial cells [33]. Isobavachalcone significantly retards leukocyte adhesion to brain endothelial cell via the inhibition of TLR4 signaling [34]. Our results showed that LPS obviously enhanced both protein and mRNA levels of TLR4, while gene silencing of TLR4 inhibited the production of inflammatory mediators and adhesion molecules in LPS-treated cells. Hy effectively impeded the increased TLR4 expressions in HMEC-1 cells response to LPS. These results indicated that Hy may counteract LPS-stimulated endothelial inflammation via the blockade of TLR4. 

PPAR-γ, one of the nuclear receptors of ligand-activated transcriptional factors, plays a pivotal role in vascular endothelial function and atherosclerosis [35,36]. PPAR-γ activation is able to alleviate inflammation response in endothelial cells [37]. Pharmacological activation of PPAR-γ ameliorates the levels of TNF-α, IL-6, soluble intercellular adhesion molecule-1 (sICAM-1) and soluble vascular cellular adhesion molecule-1 (sVCAM-1) in endothelial cells [38]. In this study, we exhibited that LPS dose-dependently inhibited both protein and mRNA levels of PPAR-γ, and Hy obviously attenuated LPS-induced suppression of PPAR-γ expression. In addition, LPS-induced TNF-α, IL-1β, MCP-1 and VCAM-1 productions can be reversed by PPAR-γ agonist pioglitazone. These results hinted that Hy could suppress LPS-induced expressions of inflammatory cytokines and adhesion molecules via activating PPAR-γ. 

Our additional data showed that the pretreatment of HMEC-1 cells with pioglitazone abolished TLR4 expressions triggered by LPS. Reduction of TLR4 by siRNA remarkably restored the downregulated PPAR-γ levels in HMEC-1 cells response to LPS. Moreover, GW9662 (a PPAR-γ antagonist) blocked the pioglitazone-mediated inhibition of TLR4 in LPS-challenged HMEC-1 cells. These results further confirmed that PPAR-γ functioned as an antagonist against LPS by its interaction with TLR4. These results implied that both PPAR-γ activation and TLR4 suppression may coordinately participate in the protective effects of Hy on endothelial inflammation induced by LPS. It has been reported that PPAR-γ is believed to inhibit TLR4 expression in quiescent vascular cells, such as endothelial cells and vascular smooth muscle cells [37,39,40]. It is an interesting question why PPAR-γ activation had no obvious effect on TLR4 expression in quiescent HMEC-1 cells. We tested whether PPAR-γ activation affects TLR4 protein expression in the human umbilical vein cell line, EA.hy926 cells. It is shown that activation of PPAR-γ with pioglitazone not only abated the basal TLR4 expression in quiescent cells, but also tremendously compromised LPS-induced TLR4 expression in EA.hy926 cells. HMEC-1 cells are of the human dermal microvascular endothelial cell line, while EA.hy926 cells are established by fusing primary human umbilical vein cells with a thioguanine-resistant clone of A549 by exposure to polyethylene glycol (PEG). We speculated that the exogenous stimulation of PPAR-γ did not modulate the TLR4 expression under physiological conditions, but played an inhibitory role in the LPS-stimulated expression of TLR4 in HMEC-1 cells.

PI3K/Akt/mTOR signaling cascade is supposed to contribute to allergic airway inflammation in asthma models [41]. Silica nanoparticles evoke an inflammatory response and endothelial dysfunction associated with the activation of the PI3K/Akt/mTOR pathway [42]. Nicotine is identified to upregulate both mannose receptor and TLR4 levels via the PI3K-Akt-mTOR-p70S6 pathway in dendritic cells [43]. The ascochlorin derivative 4-*O*-methylascochlorin (MAC) prevents the differentiation of 3T3-L1 preadipocytes through the modulation of PI3K-mTOR-PPAR-γ signaling pathways [44]. In this study, we showed that LPS-challenged HMEC-1 cells had higher levels of phosphorylated Akt and mTOR, which was substantially prevented by Hy. Blockade of both PI3K and mTOR diminished the excessive expressions of inflammatory cytokines and adhesion molecules induced by LPS. Moreover, the negative interactions between TLR4 and PPAR-γ caused by LPS were retarded by the inhibition of the PI3K/Akt/mTOR signaling pathway. These results unveiled that PI3K/Akt/mTOR-dependent modulation of TLR4 and PPAR-γ might be one of the key mechanisms by which Hy antagonized LPS-mediated endothelial inflammation. Perturbation of PI3K induces TLR4 changes in macrophages and VSMCs [45,46]. However, we found that the inhibition of PI3K/Akt/mTOR did not influence the basal TLR4 and PPAR-γ in quiescent HMEC-1 cells. The results suggested that the endogenous PI3K/Akt/mTOR signaling pathway is required for the regulation of both TLR4 and PPAR-γ expressions in LPS-incubated HMEC-1 cells, but is not involved in regulating basal TLR4 and PPAR-γ levels in the normal state. 

## 4. Material and Methods 

### 4.1. Reagents and Antibodies 

Hy was purchased from Shanghai Shifeng Technology Co., Ltd. (Shanghai, China). RNAiso Plus reagent was purchased from Takara Co. (Takara, Otsu, Shiga, Japan). Cell culture supplies were purchased from Costar (Corning Inc., Cypress, CA, USA). MCDB 131, lipopolysaccharide (LPS), GW9662 and pioglitazone were purchased from Sigma Chemical Co. (St Louis, MO, USA). Antibody against glyceraldehyde phosphate dehydrogenase (GAPDH), total or phosphorylated mTOR and goat anti-rabbit IgG H&L (Alexa Fluor^®^ 488) were purchased from Abcam (Cambridge, MA, USA). Antibodies against total or phosphorylated Akt were obtained from Cell Signaling Technology (Beverly, MA, USA). Antibodies against toll-like receptor 4 (TLR4), peroxisome proliferator-activated receptor γ (PPAR-γ) and HRP-labeled goat anti-rabbit secondary antibody were purchased from Wuhan Sanying Biotechnology Co., Ltd. (Chinese branch, Wuhan, China). LY294002, rapamycin, and DAPI were obtained from Beyotime Biotechnology Research Institute (Shanghai, China). The specific primers, siRNA sequences targeted TLR4 and negative siRNA sequences were synthesized by SangonBiotech (Shanghai, China) Co., Ltd. (Shanghai, China). The concentration of inhibitors used in the present study was determined according to previous studies and our preliminary studies. All experiments were conformed to the Medical Ethics Committee of Jiangnan University. 

### 4.2. Cell Culture 

Human microvascular endothelial cells HMEC-1 were obtained from the Health and Medicine Research of French National Institute. HMEC-1 cells were cultured in MCDB 131 medium supplemented with 10% fetal bovine serum, 2 mM l-glutamine and 1× Antibiotic-Antimycotic Solution. Human EA.hy926 endothelial cells were cultured in dulbecco modified eagle medium (DMEM) medium supplemented with 10% bovine serum. These cells were incubated at 37 °C in humidified air containing 5% CO_2_. The growth medium was replaced every 2–3 days and the cells were seeded onto petri dishes or multi-well plates at a ratio of 1 to 3 upon 80% confluency. 

### 4.3. Real-Time Quantitative PCR Analysis

The mRNA levels of tumor necrosis factor-α (TNF-α), interleukin-1β (IL-1β), vascular cellular adhesion molecule-1 (VCAM-1) and monocyte chemoattractant protein 1 (MCP-1) were detected by a fluorescence quantitative LightCycler 480 Real Time PCR system (Roche, Basel, Sweden). In short, total RNA of each sample was extracted by Trizol reagent according to the manufacturer’s instructions. Equal RNA levels (0.5 μg) from each sample were used for cDNA synthesis using HiScriptQ RT SuperMix for qPCR (Vazyme, Nanjing, China). The real-time quantitative PCR was conducted using ChamQ^TM^ SYBR^®^ qPCR Master Mix (Vazyme, Nanjing, China). The relative quantification of gene expression was reported as a relative quantity to the control value by using 2^−ΔΔ*C*t^ methods. GAPDH was used as an internal control for each sample. The sequences of primers are listed in the supplemental table (Table S1).

### 4.4. Western Blot Analysis 

The stimulated cells were washed by cold phosphate-buffered saline (PBS) three separate times, and then lysed in radioimmunoprecipitation assay (RIPA) lysis for 1 h on ice. The cell debris was discarded after centrifugation at 13,000× *g* for 15 min. Total protein was measured using BCA Protein Assay Kit (Beyotime, Nanjing, China), and an equal amount of whole cell lysates was loaded onto sodium dodecyl sulfate (SDS)-polyacrylamide gel electrophoresis and electro-transferred onto nitrocellulose membranes (Millipore, Darmstadt, Germany). Nonspecific sites were blocked in for 1 h at room temperature before overnight incubation with indicated primary antibodies at 4 °C. The blots were then subsequently incubated with the appropriate secondary antibodies conjugated to horseradish peroxidase. Immunoreactive bands were visualized by enhanced chemiluminescence (Millipore, Darmstadt, Germany). Data were normalized to GAPDH.

### 4.5. Immunofluorescence Microscopy

Four independent experiments are conducted to measure the TLR4 and PPAR-γ expressions in HMEC-1 cells. HMEC-1 cells were preincubated with or without Hy (100 μM) for 6 h before LPS incubation for another 48 h. The collected cells were fixed with 4% formaldehyde for 30 min, and then permeabilized with 0.1% Triton X-100 in PBS for 15 min. After incubation with 10% goat serum for 30 min, they were incubated with the indicated primary antibody rabbit anti-TLR4 or PPAR-γ overnight at 4 °C. After three separate washes with PBS, cells were incubated with goat anti-rabbit IgG H&L Alexa Fluor^®^ 488 for 30 min. Nuclei were stained with 4′,6-diamidino-2-phenylindole (DAPI) after immunofluorescence staining. Immunofluorescence signals were visualized on a fluorescence microscope (80i, Nikon, Japan). The fluorescence intensity was quantified using Image J software (ver. 1.43u, National Institutes of Health, Bethesda, MD, USA). The fluorescence intensity was averaged from at least six independent visual fields for each group, and the relative fluorescence intensity was assessed as previously described [47,48]. 

### 4.6. siRNA Transfections 

HMEC-1 cells were seeded onto six wells with an initial density of 10^5^ cells/mL to form a monolayer on the day before the transfection. HMEC-1 cells at 30–40% confluent were washed and resuspended in fresh medium without antibiotics and transfected separately with siRNA-TLR4 (100 nM), scramble (control) siRNA (100 nM) by using Lipofectamine 2000 (Invitrogen, Carlsbad, CA, USA), following the manufacturer’s protocols. After 6 h of incubation, the medium was changed to flesh medium supplemented with 10% serum and antibiotics. Cells were incubated with LPS for an additional 48 h. The siRNA sequences-targeted TLR4 were as follows: sense, 5′-GGGCUUAGAACAACUAGAATT-3′; antisense, 5′-UUCUAGUUGUUCUAAGCCCTT-3′. The control siRNA sequences were as follows: sense, 5′-UUCUCCGAACGUGUCACGUTT-3′; antisense, 5′-ACGUGACACGUUCGGAGAATT-3′. The siRNA-targeted TLR4 has been demonstrated to exhibit the most efficient silencing of TLR4, as previous reported [49]. 

### 4.7. Enzyme-Linked Immunosorbent Assay (ELISA) 

The protein levels of TNF-α, IL-1β, VCAM-1 and MCP-1 were detected by commercial ELISA kits (BOSTER, Wuhan, China) according to the manufacturer’s instructions, as previously described [50]. The blanks, diluted standards, or samples were added appropriately into coat wells in 96-well plates and horseradish heroxidase (HRP)-conjugated antibody was co-incubated at 37 °C for 30 min. The reaction system was terminated with stopped solution, and the absorbance was determined using a microplate reader (STNERGY/H4, BioTek, Vermont, Winooski, VT, USA). 

### 4.8. Statistical Analysis

All results were defined as mean ± SD. Comparisons within two groups were made by Student’s *t*-test. Statistical analysis was performed by ANOVA/Dunnet *t*-test for multiple group comparisons. A difference of *p* < 0.05 was considered statistically significant. 

## 5. Conclusions

Taken together, our results demonstrate that Hy protects HMEC-1 cells against LPS-induced inflammation by inhibiting PI3K/Akt/mTOR signaling pathways, followed by the interactive modulation of TLR4 with PPAR-γ. Hy may be a therapeutic agent for use in the treatment of endothelial inflammatory diseases such as atherosclerosis. It was noted that the 48-h timepoint of stimulating conditions in our study, however, provided enough time for secondary effects that may alter the intended analysis. The effects of Hy on LPS-induced inflammation in endothelial cells at earlier timepoints will be explored in our future studies.

## Supplementary Materials

Supplementary materials can be found at www.mdpi.com/1422-0067/18/4/844/s1.

## References

[1] Wiedemair W., Tukovic Z., Jasak H., Poulikakos D., Kurtcuoglu V. (2017). The breakup of intravascular microbubbles and its impact on the endothelium. Biomech. Model. Mechanobiol..

[2] Cines D.B., Pollak E.S., Buck C.A., Loscalzo J., Zimmerman G.A., McEver R.P., Pober J.S., Wick T.M., Konkle B.A., Schwartz B.S. (1998). Endothelial cells in physiology and in the pathophysiology of vascular disorders. Blood.

[3] Nomura J., Busso N., Ives A., Matsui C., Tsujimoto S., Shirakura T., Tamura M., Kobayashi T., So A., Yamanaka Y. (2014). Xanthine oxidase inhibition by febuxostat attenuates experimental atherosclerosis in mice. Sci. Rep..

[4] Gray S.P., Jandeleit-Dahm K.A. (2015). The role of NADPH oxidase in vascular disease—Hypertension, atherosclerosis & stroke. Curr. Pharm. Des..

[5] Han J.M., Li H., Cho M.H., Baek S.H., Lee C.H., Park H.Y., Jeong T.S. (2017). Soy-Leaf Extract Exerts Atheroprotective Effects via Modulation of Kruppel-Like Factor 2 and Adhesion Molecules. Int. J. Mol. Sci..

[6] Hertle E., Arts I.C., van der Kallen C.J., Feskens E.J., Schalkwijk C.G., Stehouwer C.D., van Greevenbroek M.M. (2016). The alternative complement pathway is longitudinally associated with adverse cardiovascular outcomes. The CODAM study. Thromb. Haemost..

[7] Steven S., Munzel T., Daiber A. (2015). Exploiting the Pleiotropic Antioxidant Effects of Established Drugs in Cardiovascular Disease. Int. J. Mol. Sci..

[8] Roy A., Srivastava M., Saqib U., Liu D., Faisal S.M., Sugathan S., Bishnoi S., Baig M.S. (2016). Potential therapeutic targets for inflammation in toll-like receptor 4 (TLR4)-mediated signaling pathways. Int. Immunopharmacol..

[9] Fu Y., Hu X., Cao Y., Zhang Z., Zhang N. (2015). Saikosaponin a inhibits lipopolysaccharide-oxidative stress and inflammation in Human umbilical vein endothelial cells via preventing TLR4 translocation into lipid rafts. Free Radic. Biol. Med..

[10] Roshan M.H., Tambo A. (2016). The Role of TLR2, TLR4, and TLR9 in the Pathogenesis of Atherosclerosis. Int. J. Inflamm..

[11] Menendez-Gutierrez M.P., Roszer T., Ricote M. (2012). Biology and therapeutic applications of peroxisome proliferator-activated receptors. Curr. Top. Med. Chem..

[12] Hamblin M., Chang L., Fan Y., Zhang J., Chen Y.E. (2009). PPARs and the cardiovascular system. Antioxid. Redox Signal..

[13] Hsueh W.A., Law R. (2003). The central role of fat and effect of peroxisome proliferator-activated receptor-γ on progression of insulin resistance and cardiovascular disease. Am. J. Cardiol..

[14] Wu K., Yang Y., Liu D., Qi Y., Zhang C., Zhao J., Zhao S. (2016). Activation of PPARgamma suppresses proliferation and induces apoptosis of esophageal cancer cells by inhibiting TLR4-dependent MAPK pathway. Oncotarget.

[15] Bae J., Chen J., Zhao L. (2015). Chronic activation of pattern recognition receptors suppresses brown adipogenesis of multipotent mesodermal stem cells and brown pre-adipocytes. Biochem. Cell Biol. Biochim. Biol. Cell..

[16] Mateu A., Ramudo L., Manso M.A., de Dios I. (2015). Cross-talk between TLR4 and PPARgamma pathways in the arachidonic acid-induced inflammatory response in pancreatic acini. Int. J. Biochem. Cell Biol..

[17] Ozawa M., Honda K., Nakai I., Kishida A., Ohsaki A. (2008). Hypaphorine, an indole alkaloid from Erythrina velutina, induced sleep on normal mice. Bioorganic Med. Chem. Lett..

[18] Mollica A., Locatelli M., Stefanucci A., Pinnen F. (2012). Synthesis and bioactivity of secondary metabolites from marine sponges containing dibrominated indolic systems. Molecules.

[19] Xu X., Guo H., Jing Z., Yang L., Chen C., Peng L., Wang X., Yan L., Ye R., Jin X. (2016). *N*-Oleoylethanolamine Reduces Inflammatory Cytokines and Adhesion Molecules in TNF-α-induced Human Umbilical Vein Endothelial Cells by Activating CB2 and PPAR-α. J. Cardiovasc. Pharmacol..

[20] Pan B., Kong J., Jin J., Kong J., He Y., Dong S., Ji L., Liu D., He D., Kong L. (2016). A novel anti-inflammatory mechanism of high density lipoprotein through up-regulating annexin A1 in vascular endothelial cells. Biochim. Biophys. Acta.

[21] Rodrigues-Diez R., Gonzalez-Guerrero C., Ocana-Salceda C., Rodrigues-Diez R.R., Egido J., Ortiz A., Ruiz-Ortega M., Ramos A.M. (2016). Calcineurin inhibitors cyclosporine A and tacrolimus induce vascular inflammation and endothelial activation through TLR4 signaling. Sci. Rep..

[22] Masat E., Gasparini C., Agostinis C., Bossi F., Radillo O., de Seta F., Tamassia N., Cassatella M.A., Bulla R. (2015). RelB activation in anti-inflammatory decidual endothelial cells: A master plan to avoid pregnancy failure?. Sci. Rep..

[23] Lu Z., Li Y., Jin J., Zhang X., Lopes-Virella M.F., Huang Y. (2012). Toll-like receptor 4 activation in microvascular endothelial cells triggers a robust inflammatory response and cross talk with mononuclear cells via interleukin-6. Arterioscler. Thromb. Vasc. Biol..

[24] Han Q.A., Yan C., Wang L., Li G., Xu Y., Xia X. (2016). Urolithin A attenuates ox-LDL-induced endothelial dysfunction partly by modulating microRNA-27 and ERK/PPAR-γ pathway. Mol. Nutr. Food Res..

[25] Mukohda M., Stump M., Ketsawatsomkron P., Hu C., Quelle F.W., Sigmund C.D. (2016). Endothelial PPAR-γ provides vascular protection from IL-1β-induced oxidative stress. Am. J. Physiol. Heart Circ. Physiol..

[26] Bent E.H., Gilbert L.A., Hemann M.T. (2016). A senescence secretory switch mediated by PI3K/AKT/mTOR activation controls chemoprotective endothelial secretory responses. Genes Dev..

[27] Kim H., Banerjee N., Barnes R.C., Pfent C.M., Talcott S.T., Dashwood R.H., Mertens-Talcott S.U. (2017). Mango polyphenolics reduce inflammation in intestinal colitis-involvement of the miR-126/PI3K/AKT/mTOR axis in vitro and in vivo. Mol. Carcinog..

[28] Abe J., Berk B.C. (2014). Novel mechanisms of endothelial mechanotransduction. Arterioscler. Thromb. Vasc. Biol..

[29] Ling S., Nheu L., Komesaroff P.A. (2012). Cell adhesion molecules as pharmaceutical target in atherosclerosis. Mini Rev. Med. Chem..

[30] Talreja J., Kabir M.H., M B.F., Stechschulte D.J., Dileepan K.N. (2004). Histamine induces Toll-like receptor 2 and 4 expression in endothelial cells and enhances sensitivity to Gram-positive and Gram-negative bacterial cell wall components. Immunology.

[31] Wang P., You S.W., Yang Y.J., Wei X.Y., Wang Y.Z., Wang X., Hao D.J., Kuang F., Shang L.X. (2014). Systemic injection of low-dose lipopolysaccharide fails to break down the blood-brain barrier or activate the TLR4-MyD88 pathway in neonatal rat brain. Int. J. Mol. Sci..

[32] Pasterkamp G., Van Keulen J.K., de Kleijn D.P. (2004). Role of Toll-like receptor 4 in the initiation and progression of atherosclerotic disease. Eur. J. Clin. Investig..

[33] Outzen E.M., Zaki M., Mehryar R., Abdolalizadeh B., Sajid W., Boonen H.C., Sams A., Sheykhzade M. (2017). LPS, but not Angiotensin ll, lnduces Direct Pro-lnflammatory Effects in Cultured Mouse Arteries and Human Endothelial and Vascular Smooth Muscle Cells. Basic Clin. Pharmacol. Toxicol..

[34] Lee K.M., Kim J.M., Baik E.J., Ryu J.H., Lee S.H. (2015). Isobavachalcone attenuates lipopolysaccharide-induced ICAM-1 expression in brain endothelial cells through blockade of toll-like receptor 4 signaling pathways. Eur. J. Pharmacol..

[35] Balakumar P., Kathuria S. (2012). Submaximal PPARgamma activation and endothelial dysfunction: New perspectives for the management of cardiovascular disorders. Br. J. Pharmacol..

[36] Lagana A.S., Vitale S.G., Nigro A., Sofo V., Salmeri F.M., Rossetti P., Rapisarda A.M., La Vignera S., Condorelli R.A., Rizzo G. (2016). Pleiotropic Actions of Peroxisome Proliferator-Activated Receptors (PPARs) in Dysregulated Metabolic Homeostasis, Inflammation and Cancer: Current Evidence and Future Perspectives. Int. J. Mol. Sci..

[37] Reddy A.T., Lakshmi S.P., Kleinhenz J.M., Sutliff R.L., Hart C.M., Reddy R.C. (2012). Endothelial cell peroxisome proliferator-activated receptor gamma reduces endotoxemic pulmonary inflammation and injury. J. Immunol..

[38] Zhang Y., Zhan R.X., Chen J.Q., Gao Y., Chen L., Kong Y., Zhong X.J., Liu M.Q., Chu J.J., Yan G.Q. (2015). Pharmacological activation of PPAR γ ameliorates vascular endothelial insulin resistance via a non-canonical PPAR γ-dependent nuclear factor-κ B trans-repression pathway. Eur. J. Pharmacol..

[39] Zhang L.L., Gao C.Y., Fang C.Q., Wang Y.J., Gao D., Yao G.E., Xiang J., Wang J.Z., Li J.C. (2011). PPARγ attenuates intimal hyperplasia by inhibiting TLR4-mediated inflammation in vascular smooth muscle cells. Cardiovasc. Res..

[40] Ji Y., Liu J., Wang Z., Li Z. (2011). PPARgamma agonist rosiglitazone ameliorates LPS-induced inflammation in vascular smooth muscle cells via the TLR4/TRIF/IRF3/IP-10 signaling pathway. Cytokine.

[41] Choi Y.H., Jin G.Y., Li L.C., Yan G.H. (2013). Inhibition of protein kinase C δ attenuates allergic airway inflammation through suppression of PI3K/Akt/mTOR/HIF-1 α/VEGF pathway. PLoS ONE.

[42] Duan J., Yu Y., Yu Y., Li Y., Wang J., Geng W., Jiang L., Li Q., Zhou X., Sun Z. (2014). Silica nanoparticles induce autophagy and endothelial dysfunction via the PI3K/Akt/mTOR signaling pathway. Int. J. Nanomed..

[43] Wang Y.Y., Hu Ch F., Li J., You X., Gao F.G. (2016). Increased translocation of antigens to endosomes and TLR4 mediated endosomal recruitment of TAP contribute to nicotine augmented cross-presentation. Oncotarget.

[44] Kim M., Cho H.J., Jeong Y.J., Chung I.K., Magae J., Chang Y.C. (2015). 4-*O*-methylascochlorin suppresses differentiation of 3T3-L1 preadipocytes by inhibiting PPARgamma expression through regulation of AMPK/mTOR signaling pathways. Arch. Biochem. Biophys..

[45] Kim S.Y., Jeong E., Joung S.M., Lee J.Y. (2012). PI3K/Akt contributes to increased expression of Toll-like receptor 4 in macrophages exposed to hypoxic stress. Biochem. Biophys. Res. Commun..

[46] Jiang D., Li D., Cao L., Wang L., Zhu S., Xu T., Wang C., Pan D. (2014). Positive feedback regulation of proliferation in vascular smooth muscle cells stimulated by lipopolysaccharide is mediated through the TLR 4/Rac1/Akt pathway. PLoS ONE.

[47] Hoque M.T., Robillard K.R., Bendayan R. (2012). Regulation of breast cancer resistant protein by peroxisome proliferator-activated receptor α in human brain microvessel endothelial cells. Mol. Pharmacol..

[48] Antonova L.V., Seifalian A.M., Kutikhin A.G., Sevostyanova V.V., Matveeva V.G., Velikanova E.A., Mironov A.V., Shabaev A.R., Glushkova T.V., Senokosova E.A. (2016). Conjugation with RGD Peptides and Incorporation of Vascular Endothelial Growth Factor Are Equally Efficient for Biofunctionalization of Tissue-Engineered Vascular Grafts. Int. J. Mol. Sci..

[49] Liu Y., Li T., Xu Y., Xu E., Zhou M., Wang B., Shen J. (2016). Effects of *TLR4* gene silencing on the proliferation and apotosis of hepatocarcinoma HEPG2 cells. Oncol. Lett..

[50] Bao M.H., Li J.M., Luo H.Q., Tang L., Lv Q.L., Li G.Y., Zhou H.H. (2016). NF-κB-Regulated miR-99a Modulates Endothelial Cell Inflammation. Mediat. Inflamm..

