# Supplementary material for: Hypaphorine Attenuates Lipopolysaccharide-Induced Endothelial Inflammation via Regulation of TLR4 and PPAR-γ Dependent on PI3K/Akt/mTOR Signal Pathway
Source: Int J Mol Sci. 2017 Apr 17;18(4):844. doi: 10.3390/ijms18040844 (PMC5412428; doi:10.3390/ijms18040844)
Supplement: Supplementary file 1 [file ijms-18-00844-s001.pdf]

Hypaphorine Attenuates Lipopolysaccharide-Induced Endothelial Inflammation via Regulation of TLR4 and PPAR-γ Dependent on PI3K/Akt/mTOR Signal Pathway

Haijian Sun, Xuexue Zhu, Weiwei Cai and Liying Qiu

Table S1. Primer for RT-PCR analysis.

Primers	Sequences (5′–3′)
GAPDH (Forward)	CCACATCGCTCAGACACCAT
GAPDH (Reverse)	CCAGGCGCCCAATACG
TNF-α (Forward)	TGCTGCACTTTGGAGTGATCG
TNF-α (Reverse)	TGTCACTCGGGGTTCGAGAAG
IL-1β (Forward)	TCCAGGGACAGGATATGGAG
IL-1β (Reverse)	TCTTTCAACACGCAGGACAG
MCP-1 (Forward)	GATGCAATCAATGCCCCAGTC
MCP-1 (Reverse)	TCCTTGGCCACAATGGTCTTG
TLR4 (Forward)	ATGAAATGAGTTGCAGCAGA
TLR4 (Reverse)	AGCCATCGTTGTCTCCCTAA
VCAM-1 (Forward)	TTGCTGACAGCTGACCTTTG
VCAM-1 (Reverse)	TTTAGGCCACATTGGGAAAG
PPAR-γ (Forward)	ATTCCATTCACAAGAACAGATCCAG
PPAR-γ (Reverse)	TTTATCTCCACAGACACGACATTCA

Note: GAPDH, glyceraldehyde phosphate dehydrogenase; TNF-α, tumor necrosis factor-α; IL-1β, interleukin-1β; MCP-1, monocyte chemoattractant protein 1; TLR-4, toll-like receptor 4; VCAM-1, vascular ellular adhesion molecule-1; PPAR-γ, peroxisome proliferator-activated receptor γ.

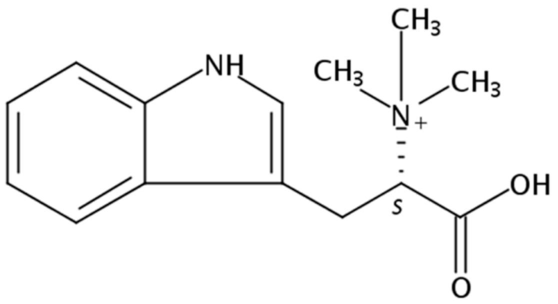

Figure S1. Chemical structures of investigated Hy.

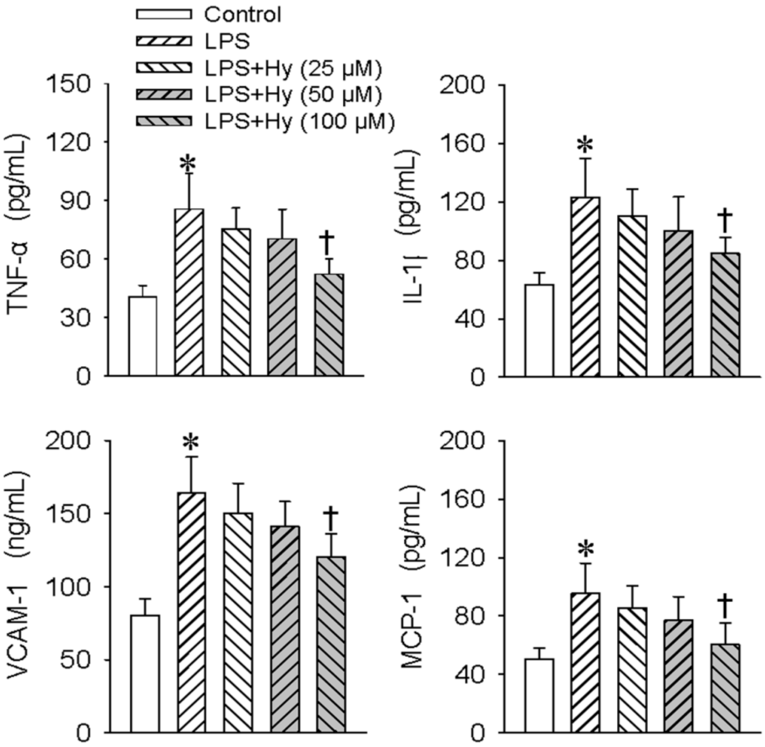

Figure S2. Effects of different doses of VH on the protein expressions of TNF-α, IL-1β, VCAM-1 and MCP-1 in HMEC-1 cells response to LPS. HMEC-1 cells were pretreated with different doses of VH for 6 h before LPS incubation for another 48 h. The protein expressions of TNF-α, IL-1β, VCAM-1 and MCP-1 were quantified by ELISA kits. Values are mean ± S.D. *$p < 0.05$ vs. Control, † $p < 0.05$ vs. LPS. $n = 6$ for each group. Hy, hypaphorine; LPS, lipopolysaccharide; TNF-α, tumor necrosis factor-α; IL-1β, interleukin-1β; VCAM-1, vascular cellular adhesion molecule-1; MCP-1, monocyte chemoattractant protein 1.

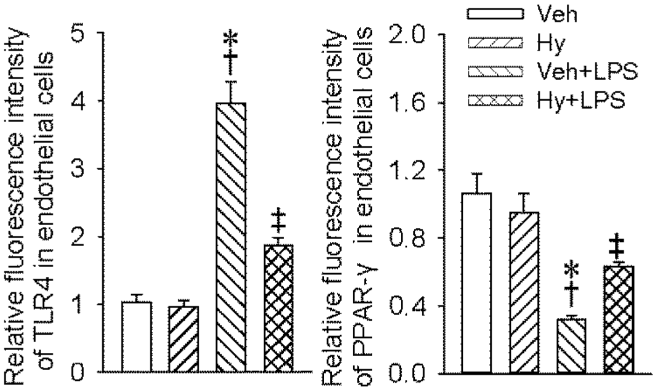

Figure S3. Average TLR4 or PPAR-γ fluorescence intensity normalized to control was obtained from four independent experiments. The mean fluorescent intensity of TLR4 (**A**) or PPAR-γ (**B**) in endothelial cells of the control group was normalized to 1.0. Values are mean ± S.D. * $p < 0.05$ vs. Veh, † $p < 0.05$ vs. VH, ‡ $p < 0.05$ vs. Veh + LPS. $n = 4$ for each group. Hy, hypaphorine; LPS, lipopolysaccharide; PPAR-γ, peroxisome proliferator-activated receptor γ.

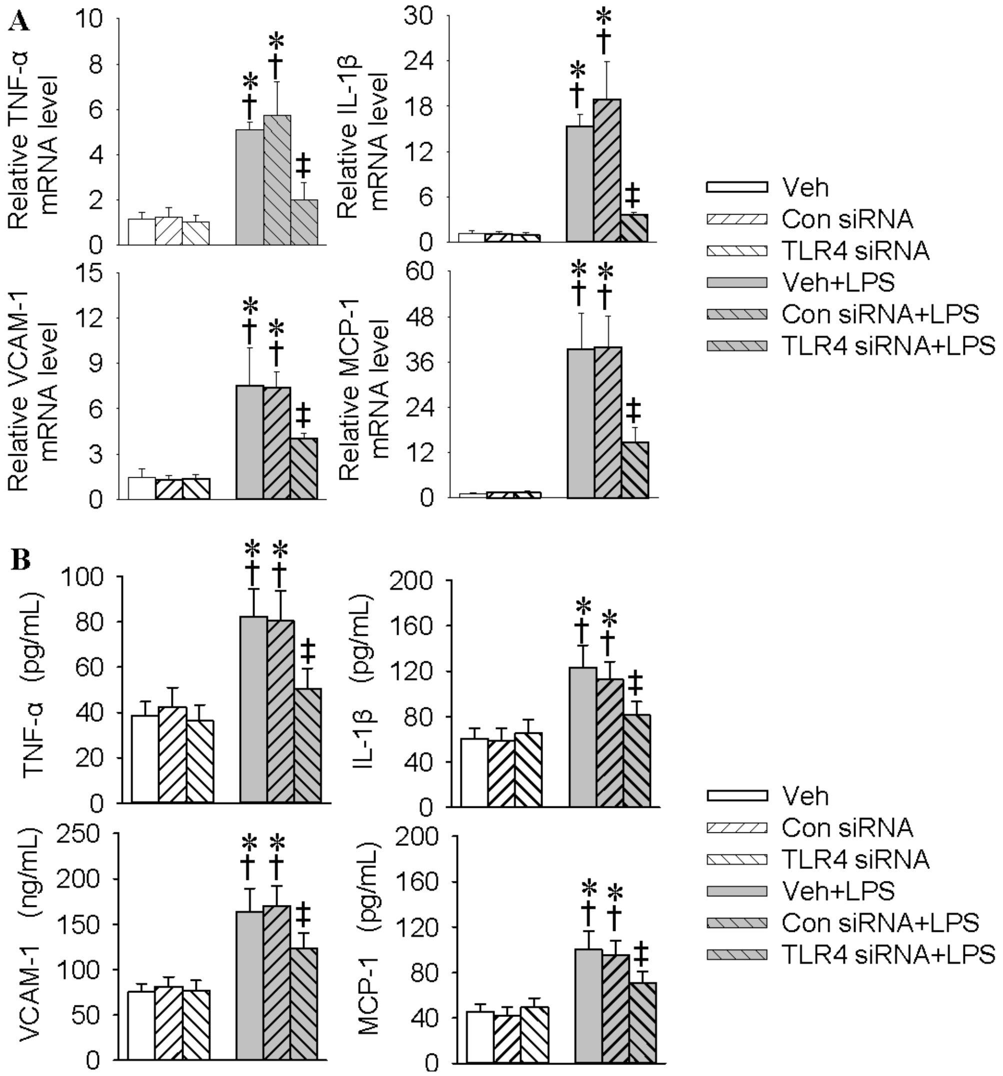

Figure S4. Knockdown of TLR4 alleviated inflammatory response in HMEC-1 cells response to LPS. The HMEC-1 cells were transfected with 100 nM Control siRNA or TLR4 siRNA for 24 h followed by LPS (500 ng/mL) stimulation for 48 h. The mRNA expressions of TNF-α, IL-1β, VCAM-1 and MCP-1 were detected by real time quantitative PCR (**A**) and ELISA (**B**). Values are mean±S.D. * p <0.05 vs. Veh, † p < 0.05 vs. Con siRNA (Control siRNA), ‡ p < 0.05 vs Veh + LPS. n = 4 for each group for PCR and n = 6 for each group for ELISA. LPS, lipopolysaccharide; TNF-α, tumor necrosis factor-α; IL-1β, interleukin-1β; VCAM-1, vascular cellular adhesion molecule-1; MCP-1, monocyte chemoattractant protein 1.

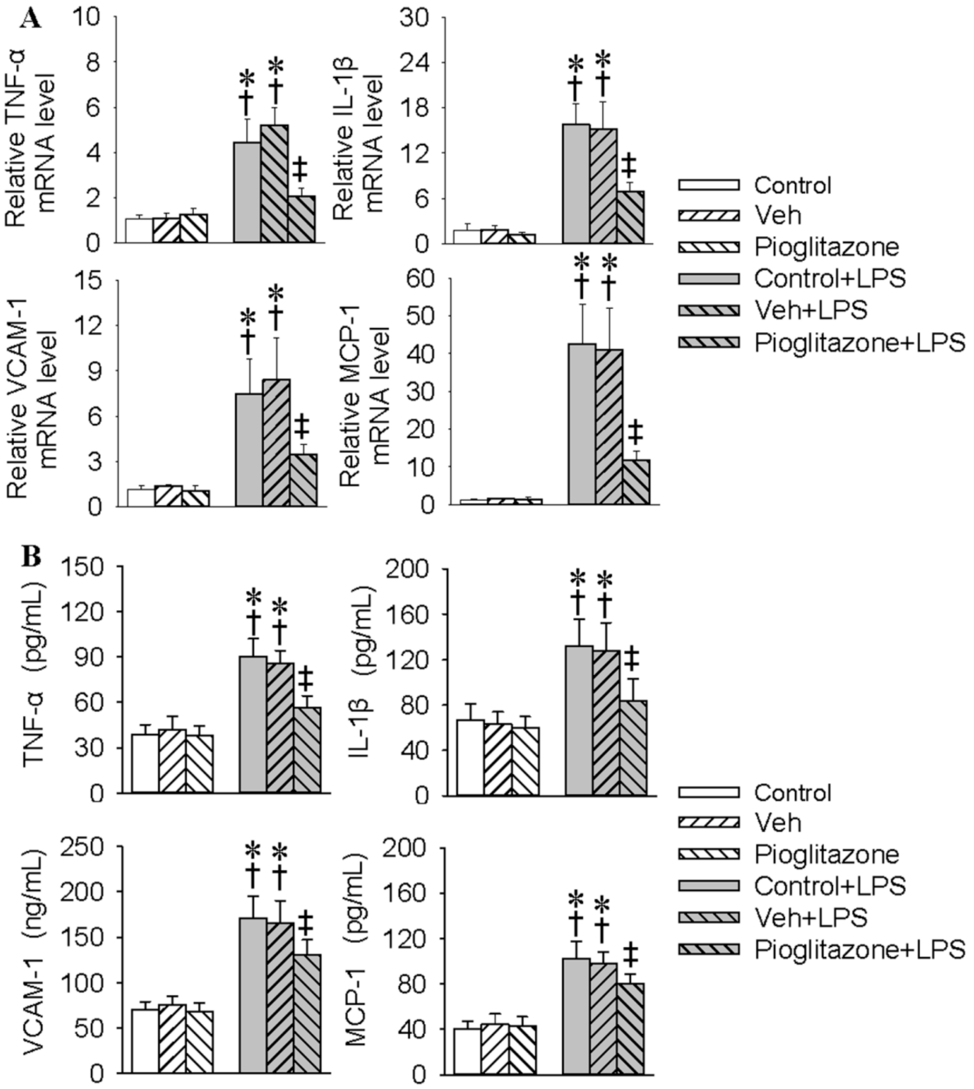

Figure S5. PPAR-γ activation ameliorated inflammatory response in HMEC-1 cells response to LPS. The HMEC-1 cells were pre-incubated with pioglitazone (20 μM) for 6 h followed by LPS (500 ng/ml) stimulation for 48 h. The mRNA expressions of TNF-α, IL-1β, VCAM-1 and MCP-1 were detected by real time quantitative PCR (**A**) and ELISA (**B**). Values are mean±S.D. **p* < 0.05 vs. Control, † *p* < 0.05 vs. Veh, ‡ *p* <0.05 vs Control+LPS. *n* = 4 for each group for PCR and *n* = 6 for each group for ELISA. LPS, lipopolysaccharide; TNF-α, tumor necrosis factor-α; IL-1β, interleukin-1β; VCAM-1, vascular cellular adhesion molecule-1; MCP-1, monocyte chemoattractant protein 1.

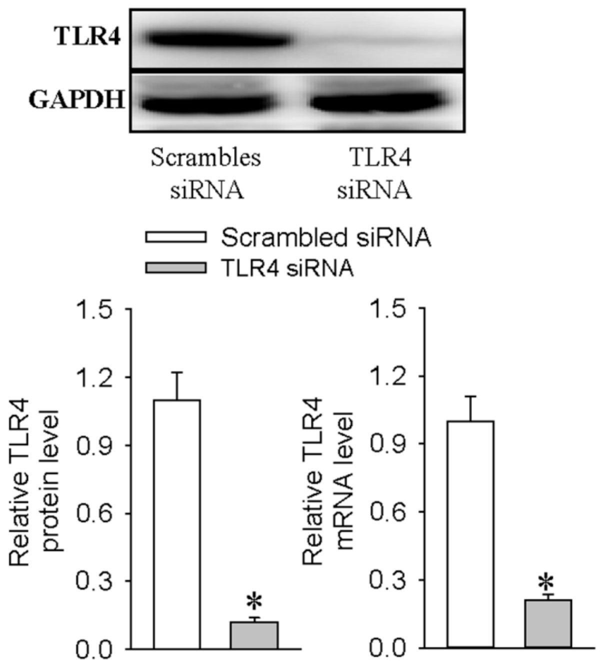

Figure S6. Knockdown of TLR4 with siRNA effectively downregulated the protein and mRNA levels of TLR4 in HMEC-1 cells. The HMEC-1 cells were transfected with 100 nM Control siRNA or TLR4 siRNA for 24 h followed by LPS (500 ng/mL) stimulation for 48 h. The protein and mRNA levels of TLR4 were measured by Western blot or RT-PCR, respectively. Values are mean ± S.D. * $p < 0.05$ vs. Scrambled siRNA. $n = 4$ for each group.

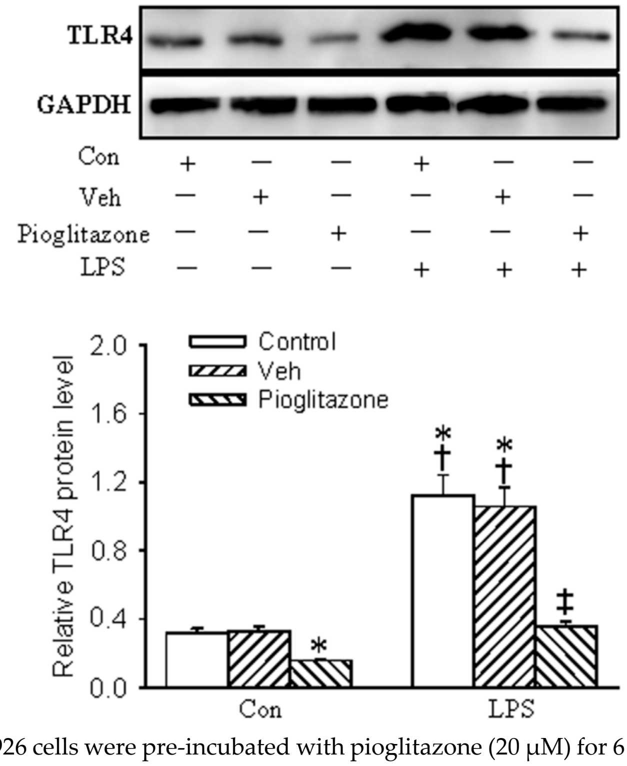

Figure S7. The EA.hy926 cells were pre-incubated with pioglitazone (20 μM) for 6 h followed by LPS (500 ng/mL) stimulation for 48 h. The protein levels of TLR4 were measured by Western blot. Values are mean ± S.D. * $p < 0.05$ vs. Control, † $p < 0.05$ vs. Veh (Vehicle), ‡ $p < 0.05$ vs. Pioglitazone. $n = 4$ for each group. Hy, hypaphorine; LPS, lipopolysaccharide; PPAR-γ, peroxisome proliferator-activated receptor γ.

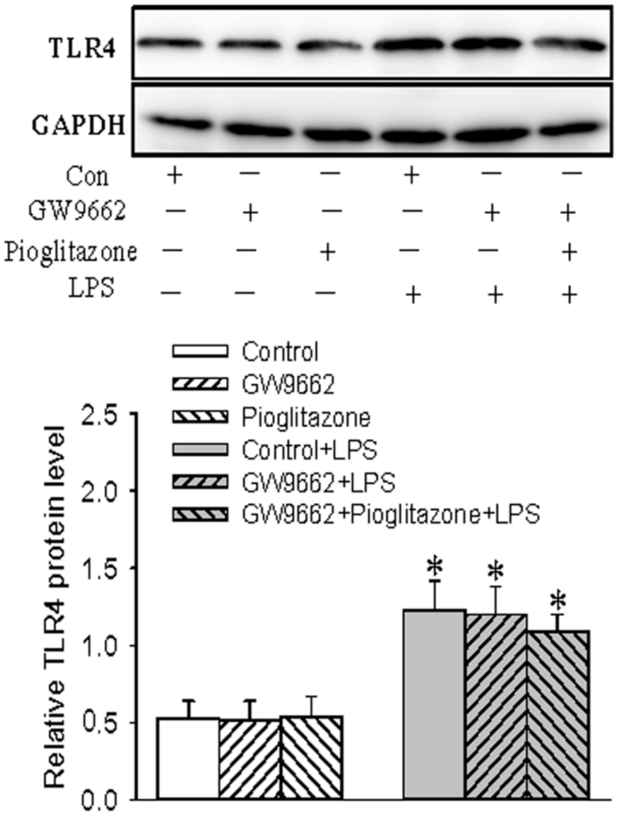

Figure S8. GW9662, a PPAR-γ antagonist, blocked pioglitazone-mediated inhibition of TLR4 in LPS-challenged HMEC-1 cells. The HMEC-1 cells were pre-incubated with GW9662 (10 μM) for 30 min, and then pioglitazone (20 μM) for 6 h, followed by LPS (500 ng/mL) stimulation for 48 h. The protein levels of TLR4 were measured by Western blot. Values are mean ± S.D. * $p < 0.05$ vs. Control. $n = 4$ for each group.

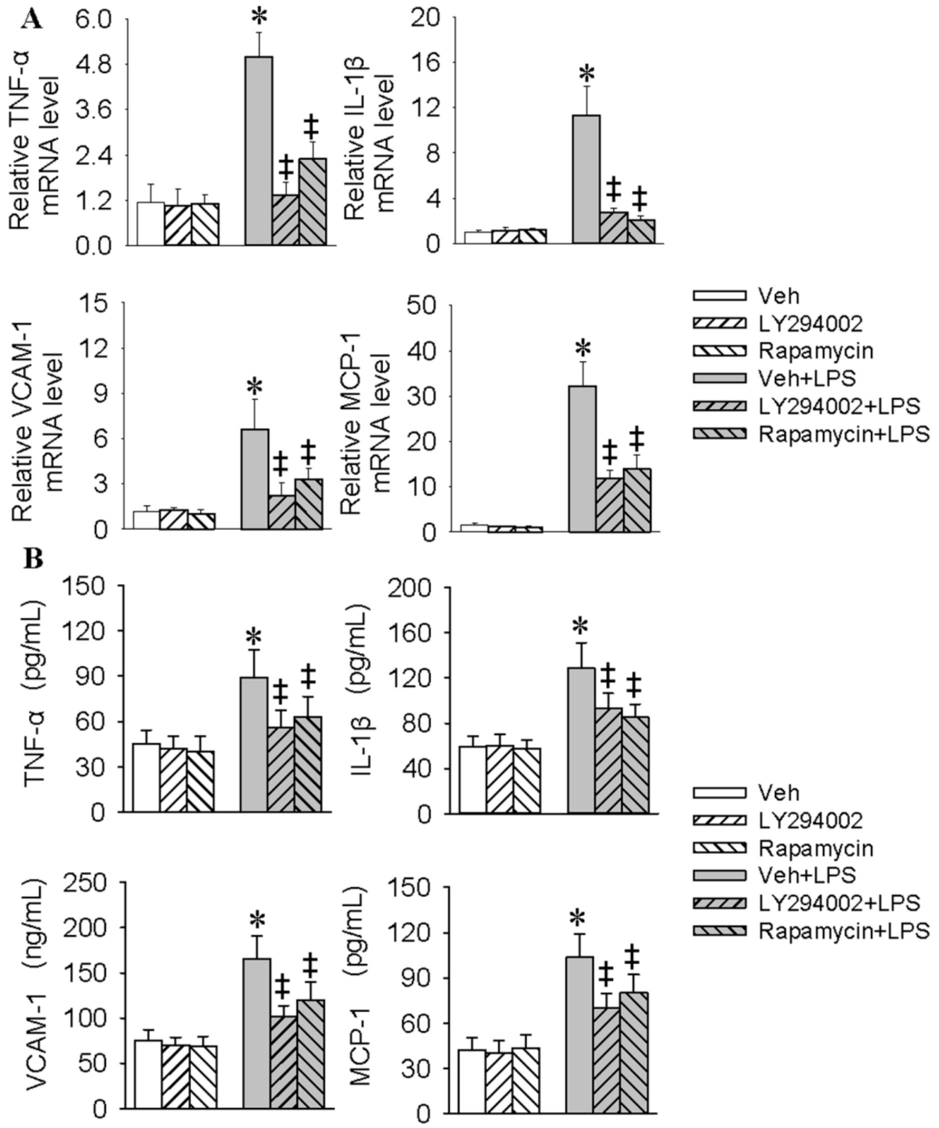

Figure S9. The HMEC-1 cells were pretreated with LY294002 (10 μM), or mTOR inhibitor rapamycin (200 nM) for 6 h before LPS incubation for 48 h. The mRNA expressions of TNF-α, IL-1β, VCAM-1 and MCP-1 were detected by real time quantitative PCR (**A**) and ELISA (**B**). Values are mean±S.D. *p<0.05 vs. Veh, ‡ p < 0.05 vs Veh+LPS. n = 4 for each group for PCR and n=6 for each group for ELISA. LPS, lipopolysaccharide; TNF-α, tumor necrosis factor-α; IL-1β, interleukin-1β; VCAM-1, vascular cellular adhesion molecule-1; MCP-1, monocyte chemoattractant protein 1.
